# Muscular Atrophy and Sclerodermia

**Published:** 1904-03-19

**Authors:** 


### MUSCULAR ATROPHY AND SCLERODERMIA.

The association of muscular atrophy with scleroderma has long been recognised, but it has usually been attributed to mere coincidence, or else the wasting of muscles has been thought to be secondary to the cutaneous changes, and caused by the compression of the altered skin or by the ingrowth of fibrous tissue from the skin to the muscle sheaths and to the muscle-tissue itself. Nixon[1] points out, however, that several cases have been recorded in recent years in which the changes in the muscles, far from following after the altered state of the skin, have preceded it, and even progressed in parts where the skin was not affected at all. The fact is, the disease has been hitherto looked at too much from the point of view of the dermatologist, and not enough attention has been paid to the muscular changes. Among the cases in which these coexist with sclerodermia there are so many points of similarity, both in the distribution and course of the affection, that the impression is conveyed that some single cause governs the changes. The disease would thus appear to form a distinct clinical entity. Nixon gives the details of an interesting case which was recently in the Royal Infirmary, Bristol. The patient was a man, aged 35. The first symptom complained of was weakness in the arms and legs, which progressed until he could no longer put on his coat. The skin became bronzed to a degree that suggested the possibility of Addison's disease. The skin of the face was yellow, dry, glossy, and giving the idea of being stretched to its utmost. Even more striking than the cutaneous alterations was the muscular wasting, the muscles of the shoulder being most affected; in some faradic irritability was absent. The general health was good, except that the patient was easily tired.

It is clear that in this case the atrophy of muscles did not depend upon the cutaneous changes; in the majority of muscles which had degenerated there was no mechanical restraint exercised upon their movements by sclerosed skin and subcutaneous tissues, and there was no ingrowth of fibrous tissue spreading down from the deeper layers of the integument, for in many places the skin was thin and elastic, and could be raised from the subjacent muscles without difficulty. Nixon remarks that the muscular atrophy was not only consistent with a central cause, but from its distribution and progress could scarcely be associated with any other.

[1] Bristol Med.-Chir. Jour., Dec. 1903.

